# Real-Time Assessment of E-Cigarettes and Conventional Cigarettes Emissions: Aerosol Size Distributions, Mass and Number Concentrations

**DOI:** 10.3390/toxics7030045

**Published:** 2019-08-30

**Authors:** Spyros Lampos, Evangelia Kostenidou, Konstantinos Farsalinos, Zoi Zagoriti, Aristeidis Ntoukas, Konstantinos Dalamarinis, Panagiotis Savranakis, George Lagoumintzis, Konstantinos Poulas

**Affiliations:** 1 Department of Pharmacy, Laboratory of Molecular Biology & Immunology, University of Patras, 265 04 Rio-Patras, Greece; 2 Institute of Chemical Engineering Sciences, ICE-HT, 265 04 Patras, Greece; 3 Institute for Research and Innovation NOSMOKE.TEAM, Patras Science Park SA, Stadiou Str., 265 04 Rio-Patras, Greece

**Keywords:** aerosols, conventional cigarettes, e-cigs, emissions, PM_1_

## Abstract

Cigarette smoke is a complex mixture of chemical compounds which are emitted during the processes of tobacco combustion. Electronic cigarettes (e-cigs) are expected to produce less harmful compounds due to the absence of tobacco leaf combustion. However, potential risks of the passive exposure to the aerosol exhaled by e-cig users have been raised in the last decade. In this study, the aerosols with diameter less than 1 μm (PM_1_) produced by vaping of various e-cig liquids were compared to those generated by smoking conventional cigarettes in real time. The mass and number concentration along with the number size distribution were measured in a closed room of 35 m^3^ volume. Our results showed that aerosols emitted from e-cig liquids had a different profile compared to those from conventional cigarettes. Although e-cigs initially produced higher particle mass and number concentrations, their emissions had much shorter lifetime of approximately 10–20 s, in comparison with the conventional and hand-rolling cigarette particulate emissions which had a dissipation time of approximately 1.4 h in a 35 m^3^ room. E-cigs emitted aerosols which volatilized rapidly, as they probably consisted almost only of propylene glycol and/or vegetable glycerin.

## 1. Introduction

Smoking is the first of the 10 major risk factors that are responsible for severe diseases in financially developed countries and is also the second cause of death globally [1]. Cigarette smoke is a complex mixture containing over 8000 chemicals, many of which have been reported as bioactive substances generated during the processes of tobacco combustion [2]. The electronic cigarette (e-cig) is known as one of the most popular alternatives to tobacco cigarette [3]. It is an electronic device designed to vaporize a mixture of nicotine, propylene glycol (PG), vegetable glycerin (VG) and other chemical compounds [4]. A typical e-cig is mainly composed of a mouthpiece, a liquid tank, a heating resistance and a battery [5,6].

All e-cigs work on the same operating principle: electrical current is delivered to the resistance, by either automatic or manual activation of the battery, and the liquid contained in the tank is evaporated and immediately condensed in a fine mist of liquid droplets (aerosol) [5,6]. Because of the absence of tobacco combustion, e-cigs are considered to be less harmful than tobacco cigarettes [3], although the exact level of risk reduction has not been accurately quantified through long-term epidemiological studies. E-cig emissions and their possible consequence on human health have been only studied for the past 10 years. Thus, their physical and chemical characterization is of crucial importance.

Mainstream cigarette smoke aerosol consists of gas-phase constituents and particulate matter. The chemical composition of the particulate phase includes polycyclic aromatic hydrocarbons, tobacco-specific nitrosamines, phenol and nicotine, whereas the vapor phase comprises carbon monoxide, carbonyls, formaldehyde, acrolein, nitrogen oxides, etc. [2]. It has been shown that e-cig vapor contains toxic, volatile organic and inorganic compounds (e.g., aldehydes and metals), as well as fine particulate matter emissions, but in notably lower concentrations compared to conventional cigarettes and the corresponding secondhand tobacco smoke [7,8,9,10,11,12,13,14,15]. Some studies indicated that the aerosol particle size and mass concentration generated from e-cigs may be similar to that of conventional tobacco cigarette [16]. The majority (~85%), though, of conventional cigarette smoke arises from side stream emissions generated during static cigarette smolder between puffs, which is absent in e-cigs, making it safer for “passive vapers” [17]. The concentration of the exhaled e-cig aerosol tends to decrease rapidly when diluted in environmental air [12]. The e-cig aerosol size ranges widely from 24–36 nm to 250–450 nm [18,19] and it can either have bimodal [20] or monomodal particle size distribution [14,16,21,22].

As the popularity of e-cigs expands, it is becoming important to further investigate patterns and levels of toxicants emitted from e-cigs. In the present study, we characterized the particles and determined the PM_1_ emissions from seven different e-cig liquids and two conventional cigarettes commercially available in the market. The experiments took place in a specially designed laboratory room (closed and without windows) with an area of ~12 m^2^ and a volume of 35 m^3^ for the real-time measurement of the generated suspended particles. 

## 2. Materials and Methods 

### Real-Time Measurement of Aerosols in a Closed Room

The experiment was conducted in a clean laboratory room without windows in the Laboratory of Molecular Biology and Immunology (Department of Pharmacy, University of Patras). The room used in this study had a volume of ~35 m^3^ and an area of ~12 m^2^. It was equipped with upper air return outlets and lower air supply outlets. Fresh air supply was set at 2 L s^−1^. The air did not recirculate through a filter. There was no visible inversion layer in the room neither during vaping nor during smoking. Room temperature was maintained at 22–25 °C. The average relative humidity of the room ranged from 25 to 35% over the duration of the study. There were no additional materials, e.g., carpet or window curtains.

A scanning mobility particle sizer (SMPS, classifier model 3080, TSI, Saint Paul, MN, USA) composed by a differential mobility analyzer (DMA, model 3081, TSI) and a condensation particle counter (CPC, model 3787 TSI) provided the size number distribution in the range of 18–947 nm and, assuming spherical particles, the volume distribution was also calculated. The SMPS was operated at a sheath flow rate of 2 L min^−1^, while the sample flow rate was 0.2 L min^−1^, and the sampling time resolution was 3 min. However, in most experiments only the CPC was used and measured the total PM_1_ aerosol number concentration with a flow rate of 0.6 L min^−1^ and a sampling time resolution of 10 s. A DustTrak (model 8553, TSI) measured the PM_1_ mass concentration of aerosols in real time with a flow rate of 3 L min^−1^ and a sampling time resolution of 10 s. The aerosol was dried prior to sampling using a diffusion dryer with silica gel. The inlet of the diffusion dryer was located in the middle of the room, ~1.2 m above the floor.

We used a SMOK XPRO M65 e-cig device (Shenzhen Ivps CO., Ltd., Shenzhen, China) (set at 6 W and R = 2.2 Ohm) and five different e-cig liquids, both commercial and laboratory-synthesized (Table 1).

We simulated the vaping processes using a 60 mL syringe (PVC). We performed at least 15 puffs of 45 mL. In addition, we conducted two experiments with a conventional and a hand-rolling cigarette brand using a smoker volunteer, who was asked to smoke one complete cigarette. Each vaping session lasted 20–30 min, while each smoking session lasted 10 min. The duration of each puff was 4 s, and there was a 30 s interval between puffs, with no deep drags or prolonged lung hold. The syringe or the volunteer was at least 1 m away from the diffusion dryer inlet.

Before each experiment, the room was vented (by keeping the door open for ~30 min), and then (with the door closed) the aerosol concentrations were allowed to reach baseline levels for 40–45 min. During this period, we measured aerosols’ levels inside the room, in order to set a baseline before starting each experiment. During both the background and the vaping/smoking measurements, there were constantly three persons inside the room. No drinks or foods were allowed. Exit and re-entry in the room were limited to minimize airflow disturbance.

## 3. Results

### E-Cigs Particulate Matter Emissions in a Closed Room

**Figure 1:**
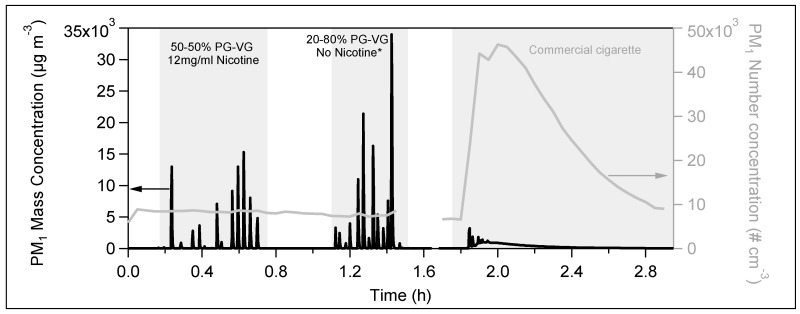
Time series of PM_1_ mass concentration as measured from DustTrak (black line) and number concentration as seen by the scanning mobility particle sizer (SMPS) (grey line) produced from two e-cig liquids and one conventional cigarette over time (the star indicates that the liquid was generated in the laboratory) during the measurements inside a 35 m^3^ closed room. The time resolution of the SMPS (3 min) was too long to capture the e-cig emissions.

Figure 1 shows the mass concentration as seen by the DustTrak and the number concentration as measured by the SMPS for two e-cig liquids (50–50% PG–VG and 20–80% PG–VG) and a commercial cigarette. E-cigs instantly produced a high aerosol mass concentration reaching more than 3000 µg m^−3^. However, these particles were volatilized in the following 10–20 s. This is probably because most of the vapor produced during vaping consisted of PG and/or VG, which are quite volatile compounds. The time resolution of the SMPS (3 min) did not allow to capture these spikes, thus for the next cases instead of the SMPS, we used only the CPC. The number size distributions (dNdlogD_p_), versus the time and the volume size distributions (dVdlogD_p_), versus the time during the vaping (not shown) did not change, indicating that the emitted particles did not form agglomerations but they were rather evaporated.

After well mixing, the maximum mass concentration of a whole conventional cigarette was 990 µg m^−3^ (black line at ~1.9 h in Figure 1). Contrary to the e-cig, the conventional cigarette emissions are much less volatile, and thus most of the particulate matter remains in the particle phase. These particles were homogeneously distributed in the room within 0.1 h (6 min). Multiplying by the room volume, the maximum particulate mass per cigarette was 35 × 10^3^ µg. This is a huge amount indicating possible adverse health effects.

**Figure 2:**
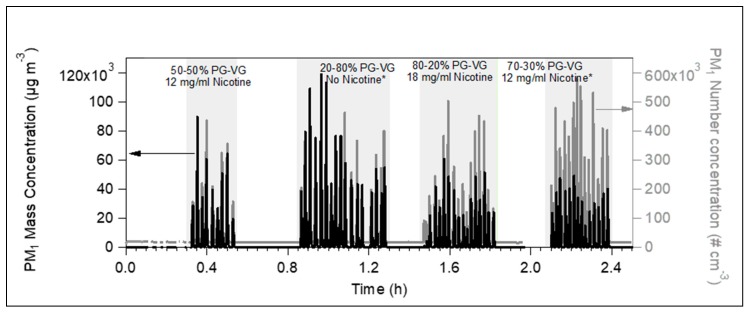
Time series of PM_1_ mass concentration as measured by DustTrak (black line) and number concentration as seen by the condensation particle counter (CPC) (grey line), produced by four e-cig liquids during the measurements inside a 35 m^3^ closed room. Independently of the e-cig liquid composition, the emissions reached high levels but they evaporated quite fast within 10–20 s.

Figure 2 illustrates the aerosol mass and number concentration, as measured by the DustTrak and the CPC, correspondingly, for four different liquids. Independently of their content in nicotine, PG or VG, all aerosols produced from the tested liquids showed the same pattern. After each puff, the particle concentrations were highly increased and then rapidly decreased. The PM_1_ mass concentration fluctuated between 15 and 120 × 10^3^ µg m^−3^, and the PM_1_ number concentration varied from 90 to 580 × 10^3^ particles cm^-3^. The short lifetime of 10–20 s did not provide enough time for the puff to be homogeneously distributed into a room of 35 m^3^, because it rapidly passed to the gas phase. Thus, the mass and number concentrations presented in Figure 1 and Figure 2 practically correspond to the amount emitted by one puff.

**Figure 3:**
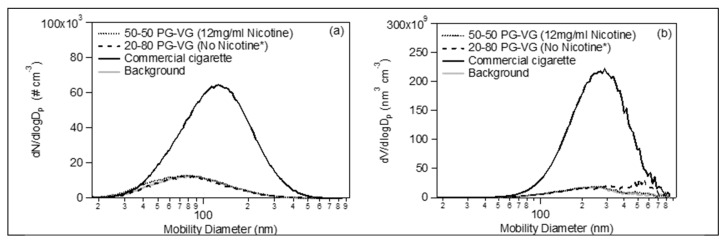
Number size distributions (**a**) and volume distributions (**b**) versus the mobility diameter produced by vaping two different e-cig liquids (black dotted and black dashed line) and by smoking a conventional cigarette (black solid line). The grey solid line represents the aerosol background inside the 35 m^3^ room. The distributions of the e-cig liquid correspond to the average of the whole vaping period of each e-cig, while the distribution of the conventional cigarette emissions is the average of 30 min after the concentration has reached the maximum value.

The average size distributions (both number and volume, Figure 3) during the vaping of the e-cig liquids—shown in Figure 1—were practically identical to those of the room’s background level. All the above indicates that the e-cigs particle emissions are very volatile and easily pass to the gas phase. The evaporation is very fast, and therefore, the indoor particle levels are not increased.

On the contrary, the conventional tobacco cigarette emitted much higher particle number, mass and volume concentration compared to the e-cigs (Figure 1). In addition, the conventional cigarette had a much longer lifetime, as the aerosol concentration in the room reached the initial levels after ~1.4 h. This is probably due to the fact that its products are much less volatile, compared to those of the e-cigs emissions. Its size number and volume distributions (Figure 3) were quite higher with respect to the background. The particles had a peak mobility diameter mode at 140 nm.

**Figure 4:**
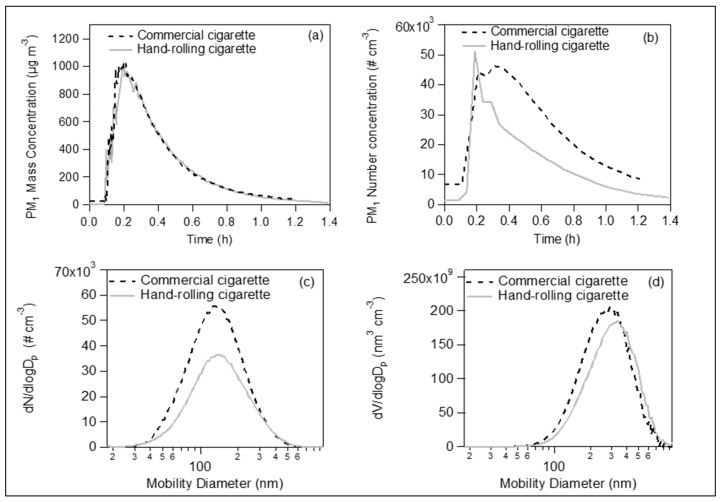
(**a**) Time series of PM_1_ mass concentration and (**b**) time series of number concentration of commercially available conventional cigarette (black dashed line) and hand-rolling cigarette (grey solid line) inside a 35 m^3^ closed room. The number size (**c**) and volume (**d**) distributions of the emitted particles had similar size ranges.

Comparing the conventional and hand-rolling cigarette, we found almost identical mass concentration profiles: one cigarette produced approximately 1000 µg m^−3^, and the removal due to deposition on the room surfaces (mainly on the walls) was achieved within 1.4 h (Figure 4a). The conventional cigarette produced 45 × 10^3^ particles cm^-3^, which was very similar to the hand-rolling cigarette (50 × 10^3^ particles cm^-3^) (Figure 4b). The number concentration, though, decreased somehow faster in the case of the hand-rolling cigarette than in that of the conventional cigarette. Their average size number and volume distributions were quite alike (Figure 4c,d).

## 4. Discussion

The use of e-cigs is constantly increasing, as they are appraised as a harmless and healthier choice compared to conventional combustion-based cigarettes. Due to the absence of tobacco leaf combustion, e-cigs are expected to produce less harmful compounds, as e-cig liquids are mainly made up of PG, VG and nicotine (the last one if provided). Recently, Shahab and colleagues reported that the long-term use of e-cigs is associated with considerably lower levels of carcinogens and toxins which are found in conventional cigarettes [23]. However, the use of e-cigs has been linked to pulmonary harm, mainly due to the relatively high concentrations of ultrafine particles (<100 nm) that can produce toxic compounds upon contact of e-cig liquids with the heating coil. Fuoco et al. recently reported that the exposure level of ultrafine particles of the mainstream aerosol can reach up to 4 × 10^9^ parts cm^−3^ [16]. However, regarding the particle size, it seems that there are some contradictions in the literature, reporting particle sizes ranging from 24 to 36 nm and from 250 to 450 nm [15,24].

Measuring particle size distribution is challenging mainly because of the relatively volatile nature of the particulate matter fraction of e-cig aerosols. In the past, most of the studies were based on off-line techniques (filter collection and extraction); however, recently developed real-time techniques are capable of revealing more important details. Herein, our aim was to measure the PM_1_ number and mass concentration along with the size distribution of several e-cig liquids and compare them with those of emissions from a commercially available conventional cigarette and a hand-rolling cigarette by advanced real-time on-line instrumentation with high resolution, under conditions that provided minimal distortion during particle sampling and delivery.

To this aim, with the aid of an SMPS, a DMA and a CPC, we found that the aerosol size distribution generated in a 35 m^3^ laboratory room by e-cigs is completely different from that of conventional tobacco cigarettes. In fact, the PM_1_ mass and number concentration of the e-cig emissions were much higher compared to those of the conventional and hand-rolling cigarette emissions. The highest concentration of nano-sized droplets was observed at the beginning of the puff. However, they evaporated within 10–20 s and did not over-stress the aerosol room concentration, as it was observed that immediately after the vaping period, the aerosol concentration reached the initial background levels. The rapid vaporization of the e-cigs emissions did not allow us to determine its size distribution in real-time conditions. On the contrary, the conventional cigarettes emitted much lower concentrations (both in mass and number), but these emissions had a much longer lifetime (1.4 h), significantly overburdening the air quality of the indoor environment. The fast vaporization of the e-cig aerosol implies that the majority of e-cig emissions are composed of volatile material, probably PG and/or VG. The phase shift from the particulate to the gas state raises concerns about the gas phase composition after vaporization and the possible additional compounds transferred to the gas phase.

Our aforementioned findings are in agreement with previous studies that showed significant amounts of nano-sized droplets detected in e-cig aerosols [15,19]. Although our study may be useful to other researchers, especially for comparisons with other reports under similar experimental conditions, we have to allude that it was limited by the absence of fast size distributions measurements regarding the e-cig distributions. Also, it does not include any chemical characterization, either for the e-cigs or for the conventional cigarettes.

## 5. Conclusions

The goal of this study was to characterize the behavior of the particles emitted by the various e-liquids and compare them to those emitted by conventional cigarettes. Concerning the particulate matter, it is clear that a room of 35 m^3^ is heavily affected by smoking conventional cigarettes but not by vaping e-cigs. However, this is probably not the same for the gas phase, which we did not study in this work. As the e-cigs emissions pass rapidly to the gas phase, the air quality would be probably affected. The next step is the determination of the e-cigs’ gas phase chemical composition and the estimation of its concentration. This is quite challenging but also very important for the evaluation of its hazardousness in humans. Our near-future studies will aim initially at the real-time characterization of e-cigs gas phase composition and at the real-time characterization of the emitted aerosol particles. The development of the real-time and high-resolution proton transfer reaction time-of-flight mass spectrometer (PTR-ToF-MS) and the high-resolution time-of-flight aerosol mass spectrometer (HR-ToF-AMS) will allow us to assess the e-cigs’ vapor mixtures in real-time order and capture the dynamic of this system. Potential future experiments will also include the study of the e-cigs emissions in an environmental chamber. The application of such techniques will contribute to e-cigs safety levels evaluation. 

## Figures and Tables

**Table 1:** The characteristics of the e-liquids and cigarette types used for the real-time measurements inside the closed room.

Exp. Number:	e-Liquids/Cigarette Type
1	80% PG–20% VG 18 mg/mL nicotine
2	50% PG–50% VG 12 mg/mL nicotine
3	80% PG–20% VG 12 mg/mL nicotine
4	70% PG–30% VG 12 mg/mL nicotine ^1^
5	20% PG–80% VG without nicotine ^1^
6	Hand-rolling cigarette
7	Conventional tobacco cigarette

^1^ Commercially available e-cigarette liquids. PG: propylene glycol, VG: vegetable glycerin.
